# Sex-specific prenatal stress effects on the rat reproductive axis and adrenal gland structure

**DOI:** 10.1530/REP-16-0097

**Published:** 2016-06

**Authors:** Cheryl J Ashworth, Susan O George, Charis O Hogg, Yu-Ting Lai, Paula J Brunton

**Affiliations:** The Roslin Institute and Royal (Dick) School of Veterinary Studies University of Edinburgh, Edinburgh, UK

## Abstract

Social stress during pregnancy has profound effects on offspring physiology. This study examined whether an ethologically relevant social stress during late pregnancy in rats alters the reproductive axis and adrenal gland structure in post-pubertal male and female offspring. Prenatally stressed (PNS) pregnant rats (*n*=9) were exposed to an unfamiliar lactating rat for 10 min/day from day 16 to 20 of pregnancy inclusive, whereas control pregnant rats (*n*=9) remained in their home cages. Gonads, adrenal glands and blood samples were obtained from one female and one male from each litter at 11 to 12-weeks of age. Anogenital distance was measured. There was no treatment effect on body, adrenal or gonad weight at 11–12 weeks. PNS did not affect the number of primordial, secondary or tertiary ovarian follicles, numbers of corpora lutea or ovarian FSH receptor expression. There was an indication that PNS females had more primary follicles and greater ovarian aromatase expression compared with control females (both *P*=0.09). PNS males had longer anogenital distances (0.01±0.0 cm/g vs 0.008±0.00 cm/g; *P*=0.007) and higher plasma FSH concentrations (0.05 ng/mL vs 0.006 ng/mL; s.e.d.=0.023; *P*=0.043) compared with control males. There were no treatment effects on the number of Sertoli cells or seminiferous tubules, seminiferous tubule area, plasma testosterone concentration or testis expression of aromatase, FSH receptor or androgen receptor. PNS did not affect adrenal size. These data suggest that the developing male reproductive axis is more sensitive to maternal stress and that PNS may enhance aspects of male reproductive development.

## Introduction

There is a wealth of epidemiological and experimental data showing that the environment to which a pregnant female is exposed can affect a range of physiological systems in the offspring, including reproductive development (Davies & Norman 2002). Exposure to stressful prenatal environments, including stressors such as extremes of temperature, light intensity, stocking density or restraint or social stressors such as mixing with unfamiliar, older or aggressive animals, can have profound post-natal effects (Ward & Weisz 1984, Meek et al. 2006, Schöpper et al. 2012, Pallarés et al. 2013). Of particular relevance and current interest are the prenatal effects of ethologically relevant stressors, such as social stress, which better reflect real-life experiences a pregnant female may encounter (for review see Brunton 2013), on offspring viability and development.

Studies conducted in a range of mammalian species have demonstrated that prenatal stress affects many aspects of reproduction including sexual differentiation of the brain, development of the reproductive organs, puberty onset, reproductive behaviour, gonad function and concentrations of reproductive hormones. The diverse prenatal stress paradigms studied, the range of reproductive end points measured and the variation in reproductive maturity of the offspring assessed in these studies make it challenging to reach any consensus. One of the more frequent conclusions, based on the effects of prenatal stress on reproductive behaviour (Weinstock 2001, Gerardin et al. 2005, Wang et al. 2006), anogenital distance (Lay et al. 2008, Pallarés et al. 2013), reproductive hormone concentrations (Ward & Weisz 1980, Ashworth et al. 2011, Pallarés et al. 2013) and both central neurotransmitter profiles (Bowman et al. 2004) and gene expression (Morgan & Bale 2011), is that prenatal stress de-masculinises or feminises the male offspring. There is also some evidence that prenatal stress masculinises the female offspring (e.g. Kaiser et al. 2003, Bowman et al. 2004), suggesting that, in general, prenatal stress reduces the difference between male and female reproductive behaviour and physiology in the offspring.

A recent re-analysis of published data describing sex differences in responses of human foetuses to adverse maternal environments supported the widely held view that developing male foetuses are more susceptible to changes in the maternal environment than female foetuses (Sandman et al. 2013). Furthermore, evidence from litter-bearing animals indicates that the foetal male reproductive axis is more sensitive to environmental change than the reproductive axis of female siblings (rats: Ward & Weisz 1984, pigs: Ashworth et al. 2011). The reasons for such sex differences are unknown, but may reflect higher growth rates in male foetuses (Aitken & Ozanne 2013), sex differences in the timing of the development of the foetal reproductive axis or in the need for pituitary support of foetal gonad development (Huhtaniemi 1989). Here, the aim is to determine whether sex differences in the response of the reproductive system to an ethologically relevant social stress imposed during pregnancy exist, as this could have important consequences for offspring management and fertility.

Various mechanisms have been proposed to explain how the physiological consequences of social stressors experienced by a pregnant female may be transmitted to her developing foetuses including alterations in the opioid system (Reznikov et al. 2005), epigenetic changes to placental gene expression (Peña et al. 2012), alterations in the maternal hypothalamic–pituitary–adrenal (HPA) axis and offspring HPA axis responsiveness (reviewed by Brunton 2013). It is generally accepted that stress elevates maternal glucocorticoid levels with some studies suggesting that the placenta of stressed mothers is less able to protect the foetus from high glucocorticoid levels (Mairesse et al. 2007) and that prenatally stressed offspring have enhanced HPA axis responses to stress (Brunton & Russell 2010). Although thermal stress experienced immediately before sample collection alters the structure of the rat adrenal cortex (Koko et al. 2004), there appear to be few, if any, studies that determined whether the structure of the adrenal gland can be programmed by prenatal stress. 

The objectives of this study are (1) to conduct an integrated assessment of the consequences of prenatal stress on reproductive status (hormone concentrations, gonad histology and gene expression) and on adrenal gland size and structure and (2) to determine whether any sex differences in the response exist. We hypothesised that, as with our earlier studies in pigs, the developing male reproductive axis would be more susceptible to prenatal stress and that the zona fasciculata of the adrenal cortex, which produces corticosterone, may be a proportionally larger component of the adrenal gland.

## Materials and methods

### Experimental animals

All procedures were performed with the approval from the University of Edinburgh Animal Welfare and Ethical Review Board and in accordance with the current UK Home Office legislation. Sprague Dawley rats were purchased from Charles River (Margate, Kent, UK). Rats were group housed (unless otherwise stated) in open-top cages and maintained on a 12-h light:12-h darkness cycle (lights on at 07:00h) under controlled conditions of temperature and humidity and with chow and water available *ad libitum*. Pregnant rats were obtained by overnight mating with sexually experienced males, and the presence of a vaginal semen plug the following morning was designated as day 1 of pregnancy. All pregnant rats were housed individually from day 14 of pregnancy and weighed daily on days 16–20. After weighing, control rats (C; *n*=9) were returned to their home cages, whereas stressed rats (PNS; *n*=9) were exposed to social stress by transferring them into the home cage of a different unfamiliar lactating rat (between days 2 and 9 of lactation) for 10min/day on days 16–20 of pregnancy inclusive, as described previously (Brunton & Russell 2010). This period of gestation coincides with foetal Sertoli cell proliferation (Orth 1982) and adrenal gland differentiation (Mitani et al. 1997). Moreover, we have demonstrated that this prenatal stress paradigm alters hypothalamo–pituitary–adrenal axis function in the adult offspring (Brunton & Russell 2010). After day 20 of gestation, rats were undisturbed throughout the remaining period of pregnancy, parturition and lactation (except for routine husbandry).

Pups were sexed on post-natal day 1 (PND1) and the combined weight of all the pups of the same sex within a litter was recorded. Dams remained within their litters until weaning on post-natal day 22. Weaned offspring were housed in groups by litter and sex. At 81±0.44 days of age, one male and one female offspring were randomly selected from each litter (to avoid within-litter effects), weighed, then killed by conscious decapitation and anogenital distance measured. Trunk blood was collected from each animal using 5% EDTA (Sigma-Aldrich) as an anti-coagulant, centrifuged at 1500 ***g*** for 10min at 4°C and the separated plasma was stored at −20°C. Adrenal glands and gonads were rapidly removed, surrounding fat was removed and the epididymis dissected from the testes and the organs were weighed. One adrenal gland and one gonad from each animal were frozen in liquid nitrogen and stored at −80°C, and the other adrenal gland and gonad were placed in methacarn solution (60% methanol, 30% chloroform and 10% acetic acid (Fisher Scientific)) for 24hours.

### Histology

After 24hours, methacarn-fixed tissues were transferred to 100% ethanol (Fisher Scientific) and stored at room temperature for 24hours. Samples were placed in histological cassettes, dehydrated by passing through graded ethanol and xylene (Fisher Scientific) and embedded in paraffin wax. From each specimen, 5 or 7µm sections were cut and placed on Polysine microscope slides (Fisher Scientific).

#### Ovarian tissue

The number of ovarian follicles and corpora lutea in ovarian sections was estimated following immunohistochemical detection of proliferating cell nuclear antigen (PCNA). Following dewaxing and heat-induced epitope retrieval (HIER) in 0.01M sodium citrate (Vector Laboratories), endogenous peroxidase activity and non-specific binding sites were blocked by sequentially incubating slides with 0.3% H_2_O_2_ (Sigma-Aldrich), in methanol and 1.5% normal horse serum (Vectastain elite ABC kit; Vector Laboratories). Two 5µm-thick sections, 100µm apart, from each ovary were incubated with mouse anti-rat PCNA (Dako UK) at 1:800 dilution or with mouse IgG (Vector Laboratories) (at the same total protein concentration) as the negative control. The slides were incubated in a humidified chamber at room temperature for 30min followed by a further 30min at room temperature with a biotinylated anti-mouse IgG secondary antibody (Vectastain Elite ABC kit; Vector Laboratories) at a dilution of 1:200 in 1.5% normal horse serum. Sections were incubated with Vectastain elite ABC reagent for 30min and with Novared peroxidase substrate (Vector Laboratories) for 5min. They were then counterstained with haematoxylin and dehydrated in a graded series of ethanol and xylene. Bright-field images were captured using a Nikon E600 microscope and a Scion cfw16012 colour camera and analysed using Image J software.

Two PCNA-stained slides from each animal were examined in order to estimate the number of primordial, transitory, primary, small pre-antral, large pre-antral and Graafian follicles, categorised as described by Lundy et al. (1999), and the number of corpora lutea. The number of large follicles and corpora lutea was counted at 1× magnification, whereas the number of smaller follicles was counted at 20× magnification.

#### Testicular tissue

Slides of testis tissue were either stained with haematoxylin (VWR, Lutterworth, UK) and eosin (Sigma-Aldrich) (H&E) or immunohistochemically stained with GATA4 (Santa Cruz Biotechnology). Bright-field images were captured using a Nikon E800XY and a Scion cfw16012 colour camera (H&E-stained slides) or E600 microscope and a Zeiss Axiocam 105 (GATA-4-stained slides). All images were analysed using Image J software.

From H&E-stained slides, the mean external area of the seminiferous tubule was determined by measuring ten seminiferous tubules per testis at 10× magnification. The numbers of spermatogonia surrounding these seminiferous tubules were determined. The number of seminiferous tubules was estimated by counting tubules in two 4× magnification images from each of four pre-determined sites on one testis slide from each animal. Spermatogenesis was categorised according to Johnsen’s score for humans (Johnsen 1970), which was modified for use in rat by Lewis-Jones and Kerrigan (1985). A mean Johnsen’s score was calculated from ten seminiferous tubules per rat.

Following immunohistological detection of the Sertoli cell marker, GATA4, the number of Sertoli cells in a defined area was determined. Dewaxing, epitope retrieval and blocking were carried out in the same manner as for the ovaries. One 7µm section from each testis was incubated with rabbit anti-human GATA4 at a dilution of 1:100 or with rabbit IgG (Vector Laboratories) at the same total protein concentration as a negative control. The slides were incubated in a humidified chamber at room temperature for 30min followed by a further 30min at room temperature with a biotinylated anti-rabbit IgG secondary antibody (Vectastain Elite ABC kit; Vector Laboratories) at a dilution of 1:200 in 1.5% normal horse serum. Sections were incubated with Vectastain elite ABC reagent for 30min and with Novared peroxidase substrate (Vector Laboratories) for 10min. They were then counterstained with haematoxylin and dehydrated in a graded series of ethanol and xylene. Sertoli cells in five images of each testis were counted at 10× magnification and their numbers related to the area of the image.

#### Adrenal tissue

Sections (5µm) of adrenal tissue were stained with haematoxylin (VWR, Lutterworth, UK) and eosin (Sigma-Aldrich). Bright-field images were captured using a Nikon E600 microscope and a Scion cfw16012 colour camera and analysed using Image J software. Three H&E-stained full adrenal cross sections per animal were assessed. The total areas of the adrenal gland, adrenal medulla, zona glomerulosa, zona fasciculate and zona reticularis of the adrenal cortex were estimated at 20× magnification.

### Hormone concentrations

Plasma concentrations of follicle-stimulating hormone (FSH) and testosterone were determined by ELISA using commercially available kits validated for rat samples (FSH: USCN-Life, Wuhan, China; Testosterone: MP Biomedicals, Santa Ana, CA, USA). All samples were analysed in a single assay. The minimum detectable concentrations were 0.0013pg/mL and 0.1ng/mL for FSH and testosterone respectively.

### Expression of aromatase and androgen and FSH receptor

RNA was isolated from a maximum of 30mg frozen testis or ovarian tissue and cDNA prepared as described by Hernandez et al. (2013). The concentrations of the isolated RNA were determined spectrophotometrically using a Nanodrop ND-1000 (Thermo Scientific). The RNA quality was assessed either by a RNA Nano chip on an Agilent 2100 Bioanalyser (Agilent Technologies) (testes) or by the High Sensitivity R6K ScreenTape System on an Agilent 2200 TapeStation (Agilent Technologies) (ovaries). The average RNA integrity number for testes tissue was 9.66 (minimum 9.3), and the average RNA integrity number equivalent for ovarian tissue was 8.93 (minimum 8.2).

The relative expression of androgen receptor (*Ar*) transcripts in testis tissue and FSH receptor (*Fshr*) and aromatase (*Cyp19a1*) transcripts in testis and ovarian tissue was measured by qPCR in a Stratagene MX3005P Real Time PCR instrument (Agilent Technologies) using Platinum SYBR Green Supermix UDG (Life Technologies) in accordance with the MIQE guidelines (Bustin et al. 2009). The primers used are presented in Table 1. The aromatase primers were designed using Primer-BLAST from NCBI. Previously published primers were used for *Ar* (Pallarés et al. 2013) and *Fshr* (Bernal et al. 2010). All gene-of-interest (GOI) primers were manufactured by Life Technologies.

**Table 1:** Primer sequences used for qPCR.

**Gene**	**Accession number**	**Amplicon size**	**Forward primer**	**Reverse primer**
Aromatase (*Cyp19a1*)	M33986	303bp	TGC GTC CTA ACA TCA CTC TAC TG	AGT TGC AGG CAC TTC CA
Follicle-stimulating hormone receptor (*Fshr*)	NM199237	151bp	TTG CTC CTG GTC TCC TTG CT	ACC TCA GTT CAA TGG CGT TCC
Androgen receptor (*Ar*)	NM012502.1	64bp	TCA CGC ACT GGC TGT ACA TTC	TGC ACC TGA CCT GGT TTT CA

Reference genes were selected using a rat-specific geNorm reference gene selection kit (PrimerDesign Ltd., Southampton, UK) following the manufacturer’s protocol. *Fshr* in ovarian tissues, *Ar* in testis tissues and *Cyp19a1* expression in both gonads were normalised using β2-microglobulin (*B2m*) and ATP synthase subunit beta (*Atp5b*). *Fshr *in testes was normalised using *B2m* and glyceraldehyde 3 phosphate dehydrogenase (*Gapdh*). cDNAs from all the samples were pooled and diluted from 1:5 to 1:5120 in nuclease-free water as standards. The cDNA of each sample and reverse transcription control was diluted at 1:25 in RNase-free water. A total of 20µL master mix (12.5µL Platinum SYBR Green qPCR SuperMix UDG, 0.1µL ROX reference dye, 2µL of each GOI primer (400 nM final concentration) or 1µL of each reference gene primer mix (240nM final concentration), 3.4µL (or 6.4µL for reference genes) nuclease-free water) was added to each well. Five microlitres of blanks (nuclease-free water), each serial dilution of standard, the samples and reverse transcription control were analysed in duplicate for each gene. PCR was carried out under the following conditions: 2min at 50°C, 2min at 95°C followed by 40 cycles of 15s at 95°C and 30s at 60°C. A dissociation stage of 1min. at 95°C and 30s at 60°C followed by data collection between 60°C and the final 30 second 95°C step was included.

These data were analysed using qBase+ software (Biogazelle, Zwijnaarde, Belgium) with target- and run-specific amplification efficiencies. The results were normalised to two reference genes and expressed relative to either the minimum value (*Ar* and *Fshr*) or a positive control (*Cyp19a1*).

### Statistical analysis

Individual pup daily weight gain between PND1 and the day of death was calculated as was the paired adrenal and gonad weight expressed as a percentage of total body weight and the anogenital distance:weight ratio. Effects of prenatal stress on litter size, total litter weight, litter sex ratios, testis morphology, and gene expression (males only) and ovarian morphology and gene expression (females only) were analysed by one-way analysis of variance using Genstat (Version 13.1 for Windows; VSN International Ltd., Oxford, UK). The effects of treatment, pup sex and their interaction on birth weight, daily weight gain, weight at death, anogenital distance, adrenal gland weight, areas of zones within the adrenal gland and hormone concentrations were analysed by two-way analysis of variance. Some variables (plasma FSH, female testosterone concentrations and ovarian aromatase expression) were log transformed to overcome scale effects. In each case, *P*<0.05 was considered to be statistically significant.

## Results

### Growth and organ size

Adrenal glands from one male PNS rat was not collected. Mean total litter size was 13.8±0.37 pups, with no differences between treatments. Within litters, 48.7% of the pups were male. Sex ratios were unaffected by treatment. Total litter weight on PND1 was significantly lower in the prenatally stressed pups compared with the controls (70.23±1.43g vs 80.92±3.21g; *P*=0.005), with both male and female prenatally stressed rats being lighter than controls of the same sex (Table 2). There was no effect of prenatal stress on post-natal daily weight gain, weight at cull, absolute anogenital distance or relative adrenal gland or gonad weight (Table 2). Anogenital distance, expressed relative to body weight, was significantly longer in PNS rats compared with control rats (0.0071 vs 0.0064, s.e.d.=0.0003cm/g; *P*=0.021) respectively. There was also a significant interaction (*P*=0.007), in which PNS reduced female anogenital distance/g but increased anogenital distance/g in the male rats. As expected, male rats grew more rapidly and were heavier at cull with heavier gonads and longer anogenital distances than their female counterparts. However, the adrenal glands accounted for a higher percentage of body weight in the female rats (Table 2).

**Table 2:** Body weight, weight change, anogenital distance and adrenal and gonad weights in prenatally stressed and control rats.

	**Control**		**Prenatally stressed**		***P***		
	Female	Male	Female	Male	Treatment	Sex	Treatment × sex
*n*	9	9	9	9			
Mean pup weight on PND1 (g)	5.81±0.35	6.15±0.38	5.03±0.21	5.32±0.23	*P*=0.011	NS	NS
Weight at death (g)	225.16±4.04	343.9±22.79	215.29±7.84	330.38±11.97	NS	*P*<0.001	NS
Weight gain between PND1 and death (g/day)	2.67±0.04	4.13±0.22	2.60±0.10	4.09±0.15	NS	*P*<0.001	NS
Paired gonad weight as % body weight	0.08±0.003	1.01±0.06	0.08±0.003	1.034±0.032	NS	*P*<0.001	NS
Paired adrenal weight as % body weight	0.040±0.002	0.023±0.001	0.038±0.002	0.022±0.002*	NS	*P*<0.001	NS
Anogenital distance (cm)	1.03±0.05	2.85±0.22	0.94±0.05	3.23±0.07	NS	*P*<0.001	0.065
Anogenital distance (cm/g)	0.0046±0.0002	0.0083±0.0003	0.0044±0.0003	0.010±0.0003	*P*=0.021	*P*<0.001	0.007

NS, not significant.

*n=8.

### Histology

#### Ovaries

The majority of ovarian follicles present in all the rats studied were primordial follicles. Prenatally stressed females tended to have more primary follicles (*P*=0.09); however, prenatal stress did not affect the number of the other types of ovarian follicles, the distribution of follicle type or the number of corpora lutea (Table 3).

**Table 3:** Abundance of ovarian follicles and corpora lutea in ovaries from prenatally stressed and control rats.

	**Control**	**Prenatally stressed**	***P***
*n*	9	9	
Primordial follicles	36.56±3.59	45.78±10.09	NS
Transitory follicles	7.33±0.97	11.0±2.55	NS
Primary follicles	3.78±0.57	5.33±0.65	0.09
Small pre-antral follicles	0.67±0.33	1.11±0.35	NS
Large pre-antral follicles	1.78±0.43	1.67±0.37	NS
Large antral follicles	8.0±0.62	8.56±1.01	NS
Corpora lutea	11.56±1.44	11.67±1.41	NS

Values are mean number of follicles or corpora lutea from two whole ovary cross sections per animal.

NS, not significant.

#### Testes

There were no significant effects of treatment on testis histology, although there was a tendency for PNS rats to have more spermatogonia (Table 4).

**Table 4:** Testis development in prenatally stressed and control rats.

	**Control**	**Prenatally stressed**	***P***
*n*	9	9	
Number of spermatogonia*^$^	36±2.45	42.33±2.81	0.109
Number of Sertoli cells/mm^2^	94.431±1.80	96.565±2.09	NS
Area of seminiferous tubule (mm^2^)^$^	1.65±0.05	1.72±0.06	NS
Johnsen’s score^$^	8.33±0.37	8.0±.33	NS
Number of seminiferous tubules^§^	50±2.31	48.72±2.61	NS

NS, not significant.

*Associated with a seminiferous tubule. Values are the means of ^$^ten or ^§^four testis sections per animal.

#### Adrenal glands

**Figure 1:**
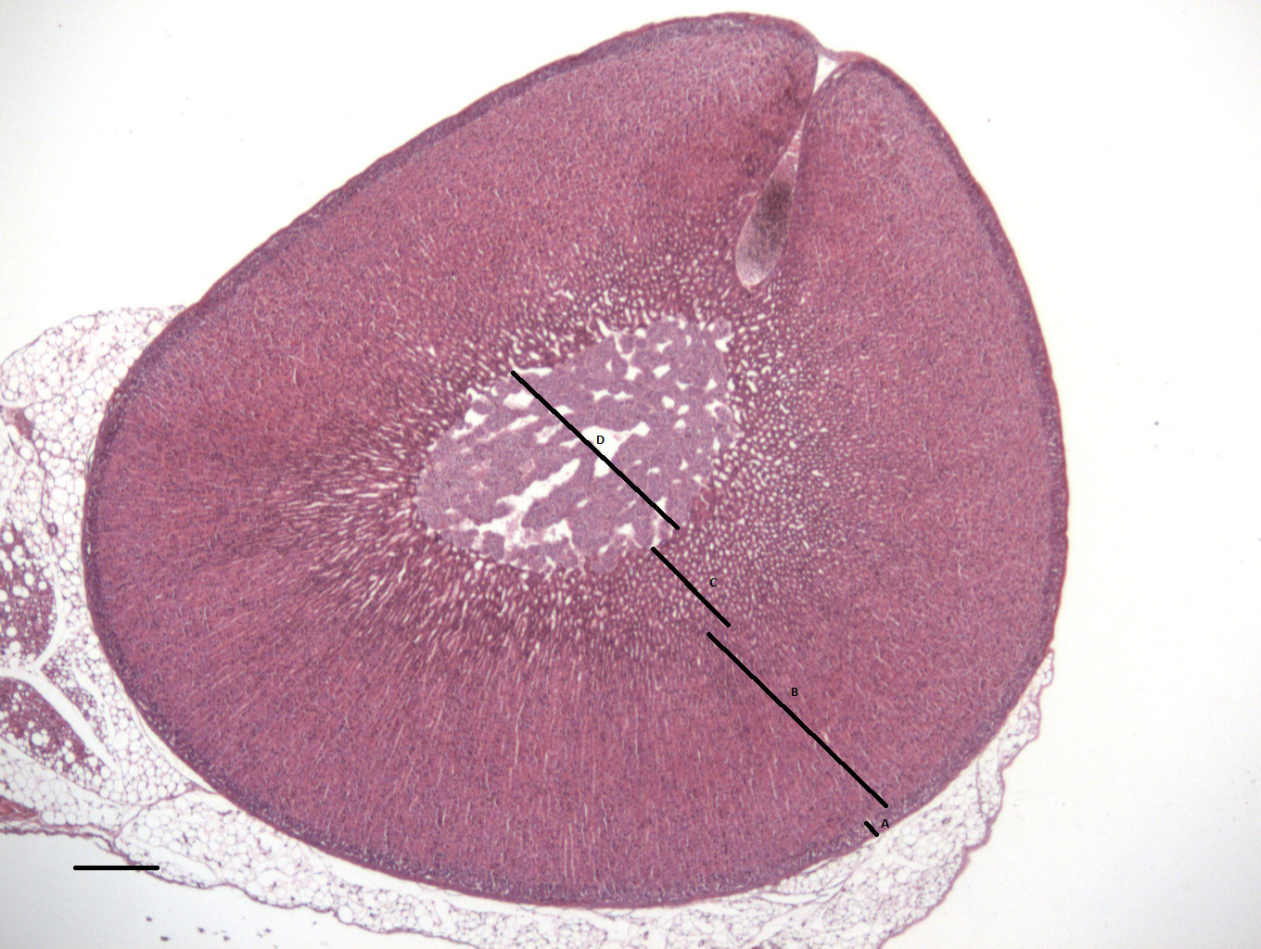
Representative section of a day 81 rat adrenal gland showing the zones measured. Adrenal medulla (A), zona reticularis (B), zona fasciculate (C) and zona glomerulosa (D). Scale bar=1mm.

There was a positive correlation between adrenal gland weight and cross-sectional surface area (*P*<0.02). Figure 1 shows a representative section of the rat adrenal gland, indicating the zones measured. There was no effect of prenatal treatment on the cross-sectional area of the adrenal gland or on the relative abundance of the zona glomerulosa, zona fasciculata or zona reticularis (Table 5). Adrenal glands from female rats had a larger cross-sectional area with relatively less zona glomerulosa and relatively more zona reticularis (Table 5). There were no significant treatment x sex interactions.

**Table 5:** Area of adrenal gland and relative area of adrenal gland zones in prenatally stressed and control rats.

	**Control**		**Prenatally stressed**		***P***	
	Female	Male	Female	Male	Treatment	Sex
*n*	9	9	9	8		
Area of adrenal gland (mm^2^)	338.66±15.9	281.92±18.24	323.0±10.27	264.37±9.51	NS	*P*<0.001
Area of zona granulosa as % total area	9.56±0.42	10.81±0.35	9.34±0.22	11.19±0.55	NS	*P*<0.001
Area of zona fasciculata as % of total area	54.20±1.58	56.72±2.12	57.50±1.63	57.06±1.49	NS	NS
Area of zona reticularis as % of total area	23.59±1.19	19.13±1.52	21.94±1.19	19.46±0.89	NS	*P*<0.01
Area of adrenal medulla as % of total area	12.28±0.76	13.29±0.89	11.28±0.89	12.71±0.93	NS	NS

NS, not significant.

#### Hormone concentrations

A plasma sample from a control female rat was lost during processing. FSH was undetectable in plasma from female rats. Male rats that were prenatally stressed had significantly higher FSH concentrations than control rats (0.05ng/mL vs 0.006ng/mL (s.e.d.=0.023, *P*=0.043) respectively). There was no significant difference in plasma testosterone concentrations between male (CON: 9.64±1.39ng/mL vs PNS: 9.71±1.73ng/mL) or female (CON: 0.41±0.05ng/mL vs PNS: 0.60±0.12ng/mL) control or PNS rats respectively.

#### Receptor expression

There was no difference between *Fshr* expression (2.29±0.25 vs 1.87±0.13 relative units) in control and PNS rat ovaries respectively. Similarly, prenatal stress did not affect testis expression of *Ar* (1.29±0.03 vs 1.27±0.06 relative units) or *Fshr* (0.86±0.02 vs 0.85±0.03 relative units) in control and PNS rats respectively.

#### Aromatase expression

There was no effect of prenatal stress on *Cyp19a1 *gene expression in testis tissue; however, ovaries from PNS females tended to have less *Cyp19a1* expression than ovaries from control females (0.547±0.10 vs 0.36±0.103 relative units; *P*=0.095).

## Discussion

This study has shown that exposure to an ethologically relevant social stress during the last week of pregnancy in the rat affected the reproductive axis of the male, but not the female, offspring. The observations that prenatally stressed male rats had longer relative anogenital distances and higher plasma FSH concentrations join the growing body of recent data (Pallarés et al. 2013, Brunton et al. 2015) indicating that some aspects of reproductive function are enhanced in sexually mature male rat offspring whose mothers were subjected to stress during pregnancy. Similar findings have been found in sheep, in which sexually mature male offspring born to mothers managed in poor husbandry conditions had larger testes with larger seminiferous tubules (Moutevelidi et al. 2015). These findings are in marked contrast to reports that suggest prenatal stress de-masculinises the male offspring (Ward & Weisz 1980, Weinstock 2001, Bowman et al. 2004, Gerardin et al. 2005, Wang et al. 2006, Morgan & Bale 2011, Pallarés et al. 2013). The reason(s) for this different response are not known; however, it is possible that variations in the stage of pregnancy when the stress was imposed, the duration, predictability and severity of the stress may alter later outcomes. For example, the work described by Bowman et al. (2004) and Pallarés et al. (2013) imposed a restraint stress three times a day for the complete last week of pregnancy, whereas the studies of Morgan & Bale (2011) involved subjecting mice to a range of stressors during early pregnancy.

Most studies investigating the effects of prenatal stress on rat reproductive development suggest that, as in this study, effects are more pronounced in the male offspring. However, a recent study by Barra et al. (2014) reported that exposure to 4°C for 3h a day throughout pregnancy in rats delayed ovarian follicular development on post-natal days 4 and 30, and delayed the onset of puberty in the female offspring. This study, which used post-pubertal rats, showed no significant alteration in the populations of ovarian follicles, although ovaries from prenatally stressed female rats tended to have more primary follicles. The tendency for ovaries from rats born to stressed mothers to have lower aromatase mRNA expression suggests that the ability to synthesise oestradiol may be compromised in these animals.

The sex differences observed in this study could reflect the more dynamic nature of male, compared with female, foetal reproductive development at the stage of gestation when the stress was imposed. Rat foetal testes develop rapidly during the last week of gestation with testicular cords formed on day 16, differentiation of Leydig cells occurring on day 17 and the first wave of Sertoli and Leydig cell proliferation occurring during late foetal life (Orth 1982). Foetal testes secrete testosterone, which reaches a prenatal peak on days 18 and 19 (Weisz & Ward 1980) and is important for post-natal reproductive development. By contrast, female rat pups are born with relatively immature ovaries (Takagi et al. 2007) that are steroidogenically quiescent until the second week of life (Weniger 1993).

Sex differences in reproductive axis responses to prenatal stress are often explained by differential effects of stress-induced alterations in the foetal HPA axis on the male and female foetal hypothalamic–pituitary–gonadal (HPG) axis (Davies & Norman 2002, Brunton 2013). However, the rat testis appears to be independent of pituitary hormone support throughout gestation (reviewed by O’Shaughnessy & Fowler 2011), whereas the responsiveness of the rat ovary to LH does not appear until the end of the first week of life, coincident with the onset of ovarian steroidogenesis (Sokka et al. 1996), suggesting that alterations in the foetal HPG axis are not likely to explain the sex differences observed.

It is generally accepted that prenatal stress programmes HPA axis responses (Brunton 2013). An earlier experiment using the same prenatal stress paradigm as in this study reported that both male and female prenatally stressed rats challenged with immune challenge or restraint stress show markedly greater ACTH and corticosterone secretion than the controls (Brunton & Russell 2010). In another study, heat-stress imposed for an hour immediately before tissue collection caused an increase in plasma ACTH and corticosterone concentrations in adult rats, which was accompanied by a reduction in the overall size of the adrenal gland and of the zona fasciculata in particular (Koko et al. 2004). This raises the question of whether prenatal stress could programme either adrenal gland size or the relative size of distinct functional zones within the adrenal gland. This study does not show an effect of prenatal stress on the size or the structure of the adrenal gland, suggesting that prenatal stress-associated alterations in corticosterone secretion are not a consequence of changes in adrenal gland structure. Rather, they appear to be a result of increased excitatory drive to the HPA axis at the level of the hypothalamus (Brunton & Russell 2010). This study confirmed that post-pubertal female rats have larger adrenal glands than males (Hatai 1913) and that sex differences in the size of the zones of the adrenal cortex exist (Majchrzak & Malendowicz 1983). Compared with male rats, female rats in this study had a smaller zona glomerulosa, the zone primarily responsible for mineralocorticoid secretion, and a larger zona reticularis, which secretes sex steroids. The significance of this finding remains to be elucidated.

Of particular interest in this study is that the prenatally stressed male rats had longer anogenital distances and higher plasma FSH concentrations. The anogenital distance reflects the migration of the genital orifice to the navel during foetal life, which is controlled by foetal testosterone concentration (Wilson & Davies 2007). Longer anogenital distances are therefore likely to reflect higher foetal testosterone concentrations. Although foetal testosterone levels were not measured in this study, other models of maternal stress in rats show an attenuation of the prenatal foetal testosterone surge (Ward et al. 2003) and, in contrast to the results of this study, a reduction in post-natal anogenital distance (Pallarés et al. 2013). Our previous studies using the same experimental paradigm found higher concentrations of total (but not free) testosterone in prenatally stressed male offspring (Brunton et al. 2015). In this study, testosterone concentrations in offspring were not significantly different between treatments. The reason for this discrepancy is not clear, but may reflect differences in the source of the plasma (trunk blood vs venous blood) between the two experiments.

The increased FSH concentrations in PNS rats suggest that their Sertoli cells would be exposed to more FSH, which along with testosterone regulates Sertoli cell function, inhibin secretion and, indirectly, spermatogenesis (Abel et al. 2008). In support of this, and although not statistically significant, the number of spermatogonia per seminiferous tubule in this study tended to be greater in the prenatally stressed males. Furthermore, a recent study reported that the male offspring of rats that were subjected to restraint stress three times a day during the last week of pregnancy had accelerated spermatogenesis both pre- and post-puberty (Pallarés et al. 2013), further supporting the concept that prenatal stress may promote male reproductive development. The physiological significance of this remains to be elucidated; however, it may reflect a strategy to increase the likelihood that offspring born in disadvantaged environments are able to reproduce.

The mechanisms by which the foetal male reproductive axis responds to stress-induced changes in maternal physiology are unclear, but could involve alterations in the relationship between maternal and/or the foetal HPA and HPG axes, epigenetic factors and/or alterations in opioids and neurosteroids and will likely depend on the duration and the severity of the stress imposed. For example, rats subjected to chronic restraint stress during the last 7–11 days of pregnancy have reduced placental expression and activity of the enzyme 11β-hydroxysteroid dehydrogenase type 2 (11βHSD2) (Mairesse et al. 2007, Peña et al. 2012), which protects the foetus from high circulating concentrations of maternal corticosterone. Peña et al. (2012) also report prenatal stress-induced changes in DNA methylation at CpG sites within the placental and foetal hypothalamic *Hsd11b2* gene promoter on embryonic day 20 and propose alterations in DNA methylation as a mechanism by which prenatal stress alters placental *Hsd11b2* expression.

In the study of Mairesse et al. (2007), day 21 foetuses carried by stressed dams did not have altered plasma corticosterone levels, but did have atrophied adrenal glands and reduced foetal ACTH, suggesting that maternally derived corticosterone was the major contributor to foetal concentrations. Male foetuses also had smaller testes; however, there was no direct evidence that this was linked to alterations in foetal or maternal glucocorticoids. Furthermore, Brunton and Russell (2010) observed no effects of prenatal stress on basal ACTH and corticosterone concentrations in 11-week-old male rat offspring. These findings, coupled with the fact that foetal rat testis (O’Shaughnessy & Fowler 2011) and ovary development (Sokka et al. 1996) is independent of pituitary gonadotrophins, suggesting that alterations in the foetal HPG axis are not likely to explain the response observed.

Other potential mechanisms should be considered. For example, observations that administration of naltrexone to stressed pregnant rats blocks the feminising effects of prenatal stress on male offspring (Keshet & Weinstock 1995, Reznikov et al. 2005) indicate that opioids may be involved in mediating the effects of stress on the reproductive axis.

In conclusion, the results of this study indicate that exposure to an ethologically relevant social stress may enhance the development of the reproductive axis in the male offspring. Further studies are required to determine whether such an intervention is associated with enhanced male reproductive performance.

## Declaration of interest

The authors declare that there is no conflict of interest that could be perceived as prejudicing the impartiality of the research reported.

## Funding

The Roslin Institute receives Institute Strategic Grant funding from the BBSRC (BB/J004316/1). Y T L was in receipt of a studentship from the Ministry of Education, Taiwan.
